# Supplementary material for: Infrared Spectroscopy of Urine for the Non-Invasive Detection of Endometrial Cancer
Source: Cancers (Basel). 2022 Oct 13;14(20):5015. doi: 10.3390/cancers14205015 (PMC9600188; doi:10.3390/cancers14205015)
Supplement: Supplementary file 1 [file cancers-14-05015-s001.zip › cancers-1932162-supplementary.pdf]

# Supplementary

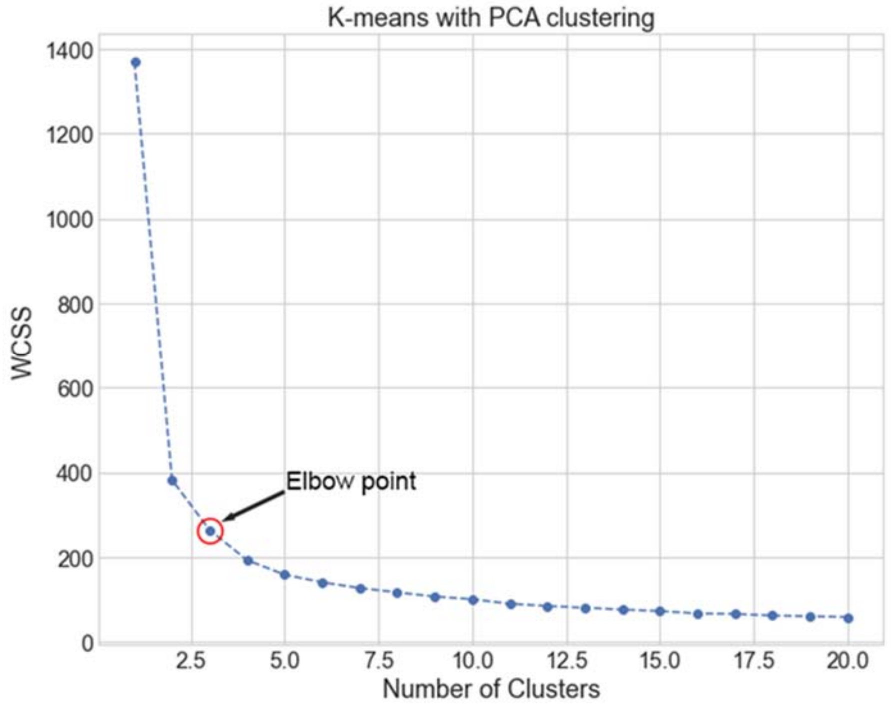


**Figure S1.** Clustering determination of PCA-K means model. The red circle illustrates the elbow point, where the optimal number of clusters where the WCSS decreases in a linear fashion.

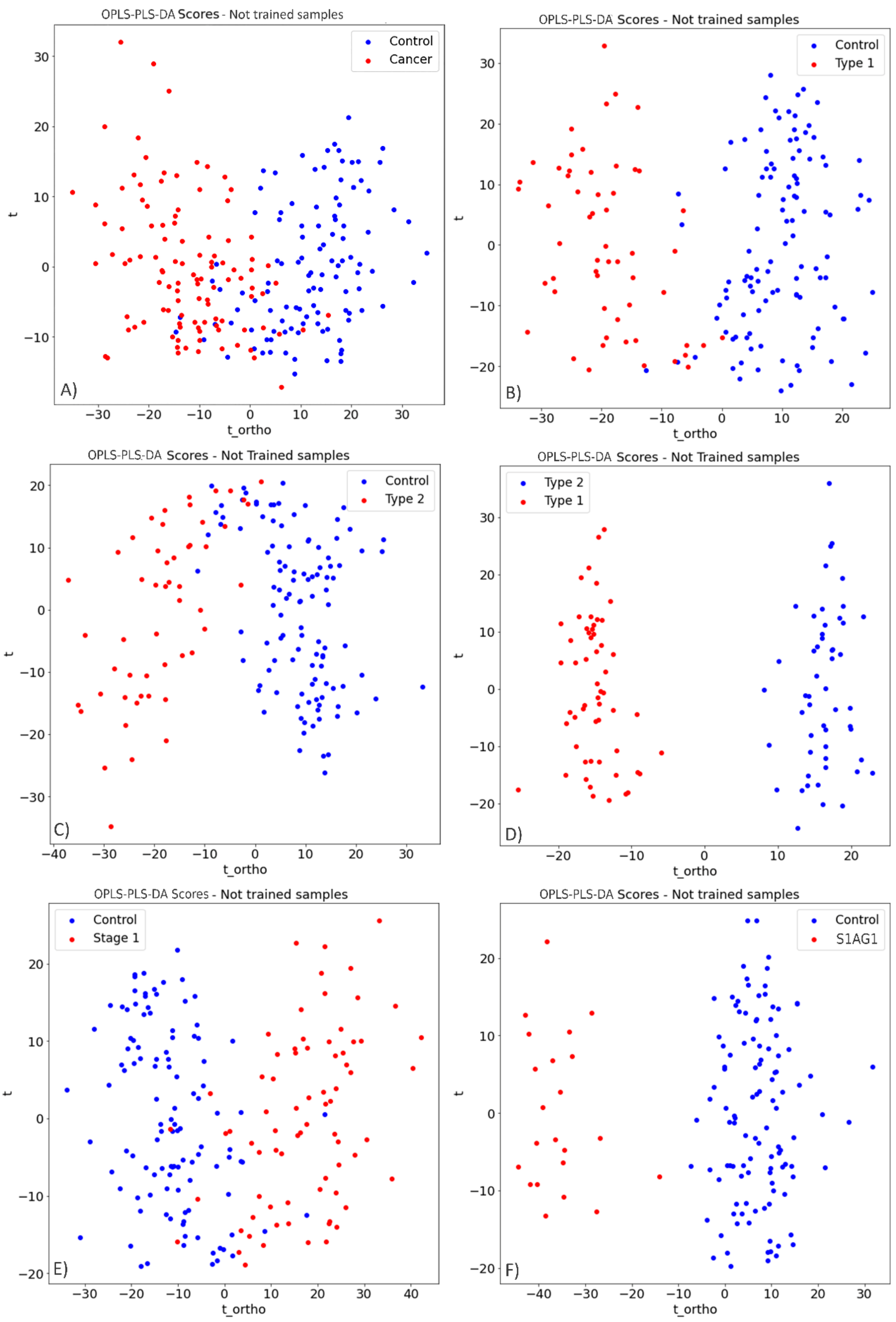


**Figure S2.** Discrimination score plots from the untrained samples processed with OPLS-PLS-DA. P-value for all discriminatory plots was $p < 0.0001$ demonstrating highly significant difference between each cluster comparison.

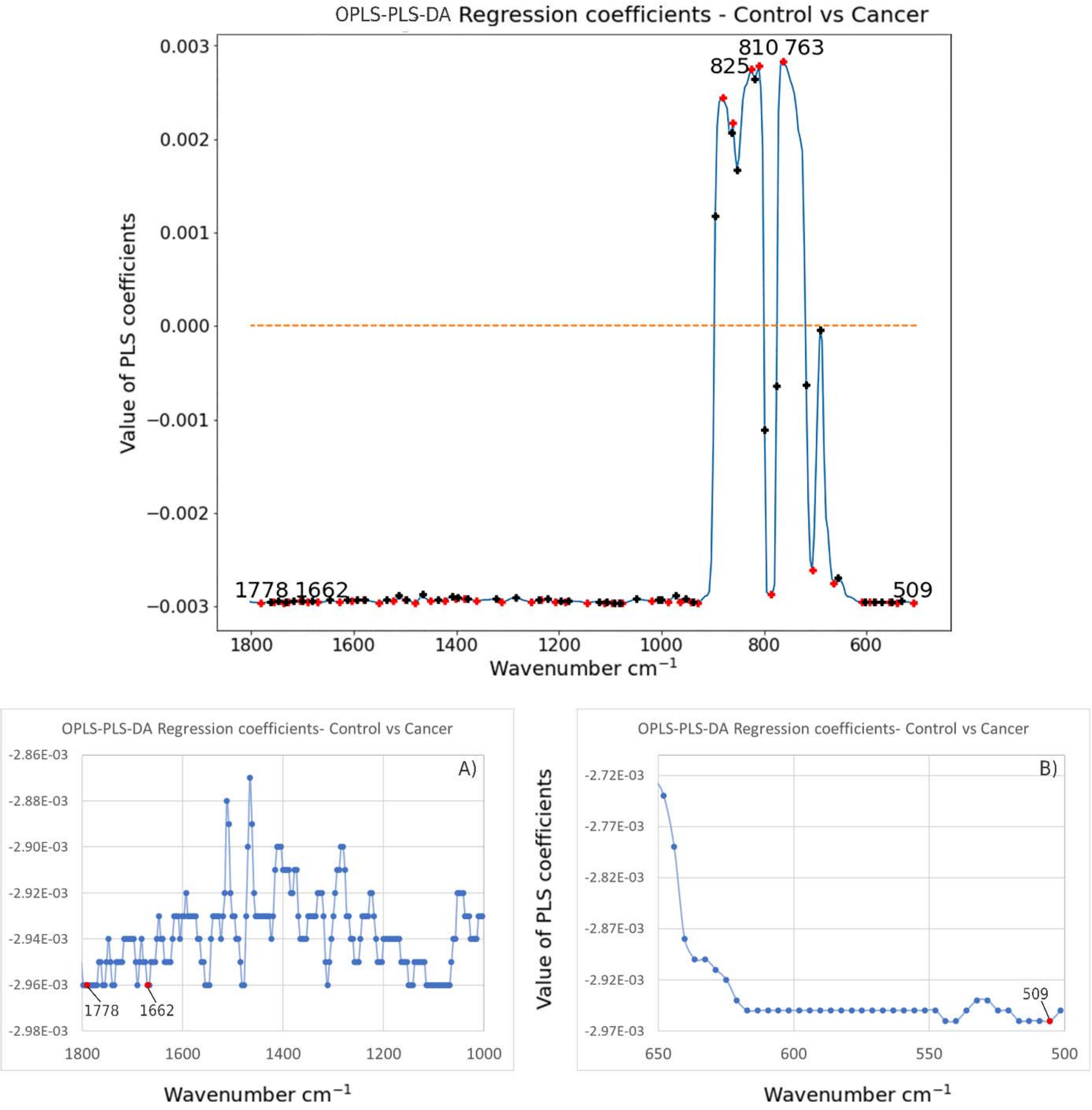


**Figure S3.** OPLS-PLS-DA Regression coefficients for Control vs Cancer. A – B figures ilustrate a close-up to the regression coefficients regions where it is not possible to discern the highest coefficient scores in positive and negative leverage. Only the highest coefficient scores are shown.

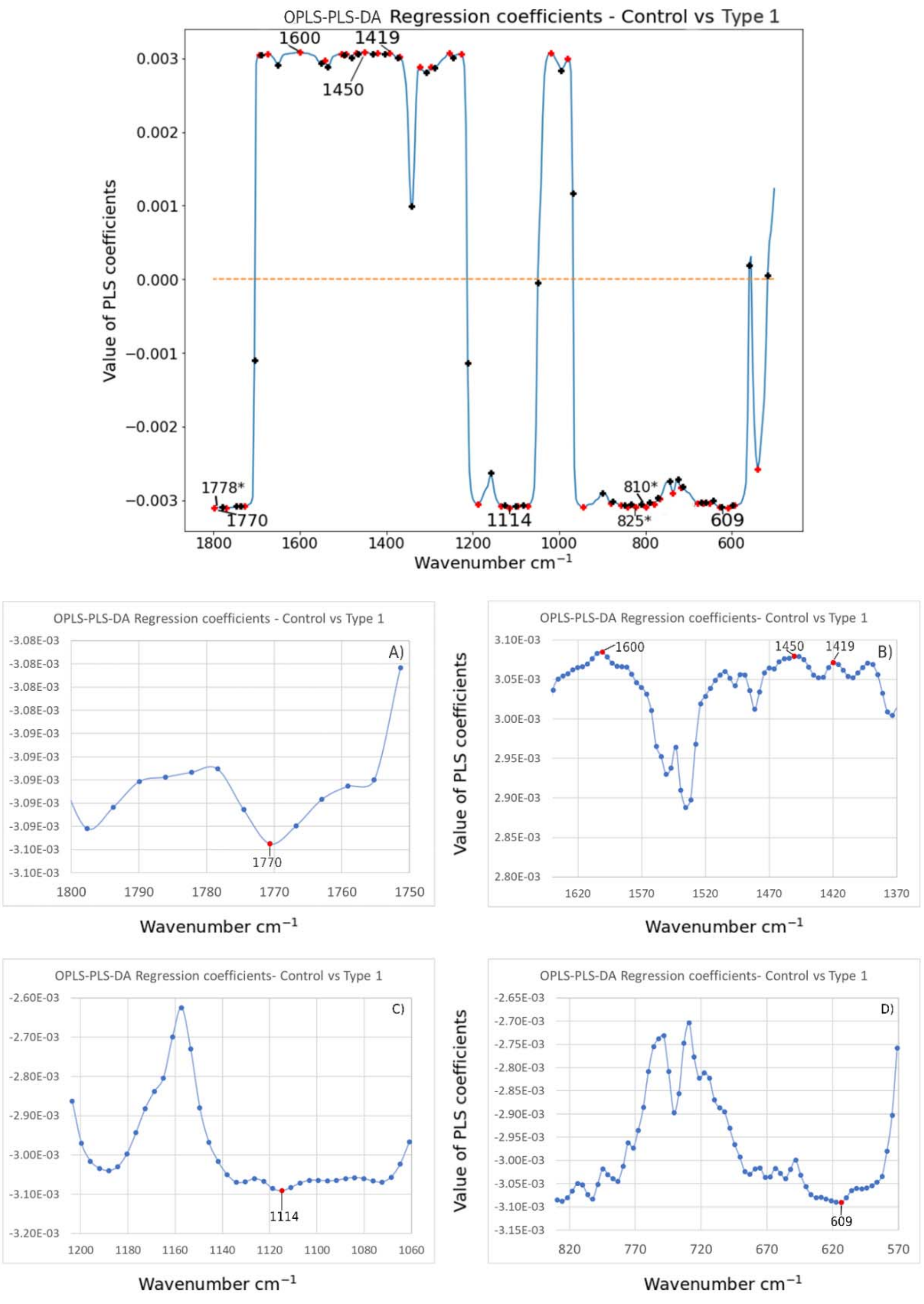


**Figure S4.** OPLS-PLS-DA Regression coefficients for Control vs Type 1. A – D figures ilustrate a close-up to the regression coefficients regions where it is not possible to discern the highest coefficient scores in positive and negative leverage. Only the highest coefficient scores are shown.

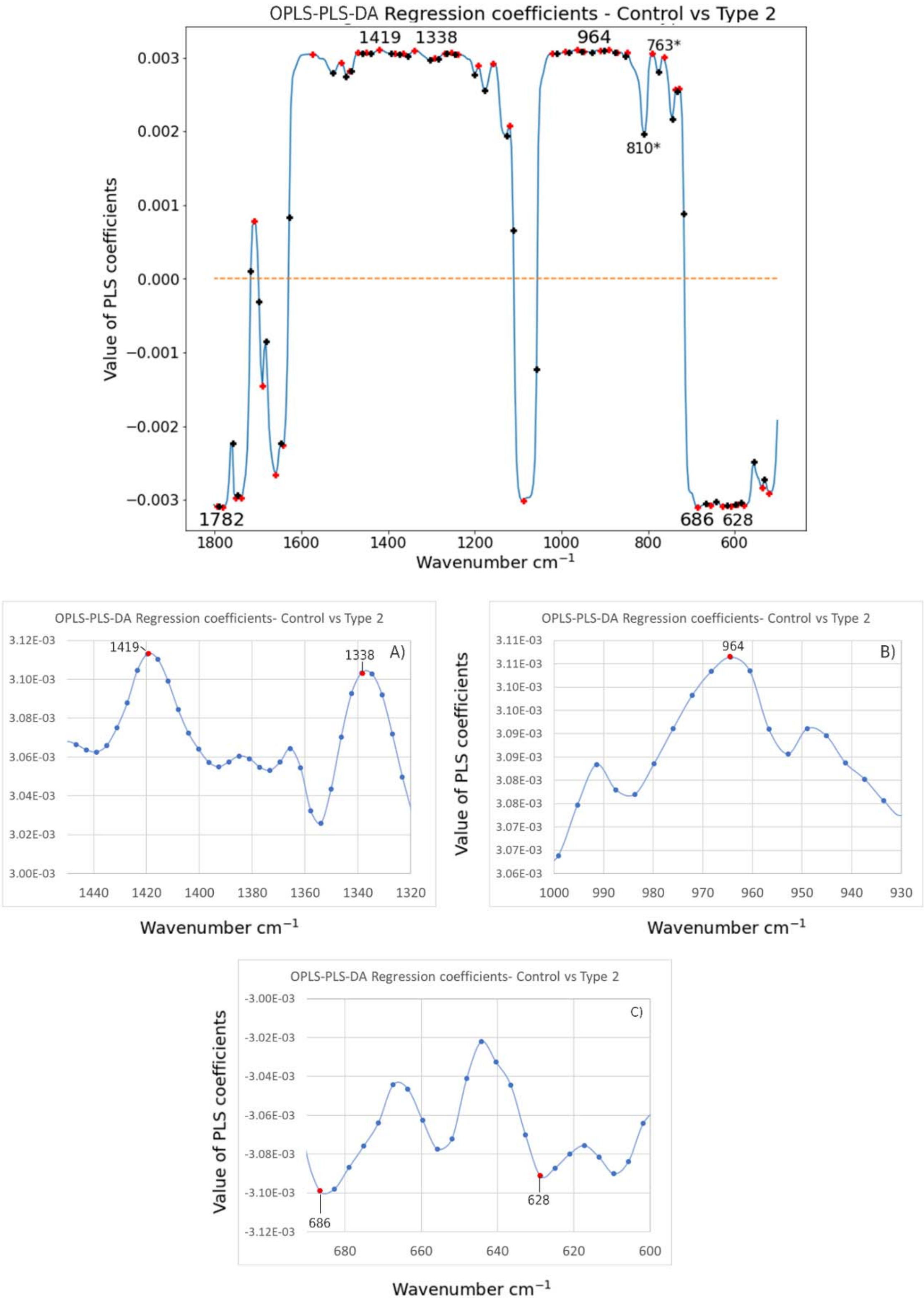


**Figure S5.** OPLS-PLS-DA Regression coefficients for Control vs Type 2. A – C figures ilustrate a close-up to the regression coefficients regions where it is not possible to discern the highest coefficient scores in positive and negative leverage. Only the highest coefficient scores are shown.

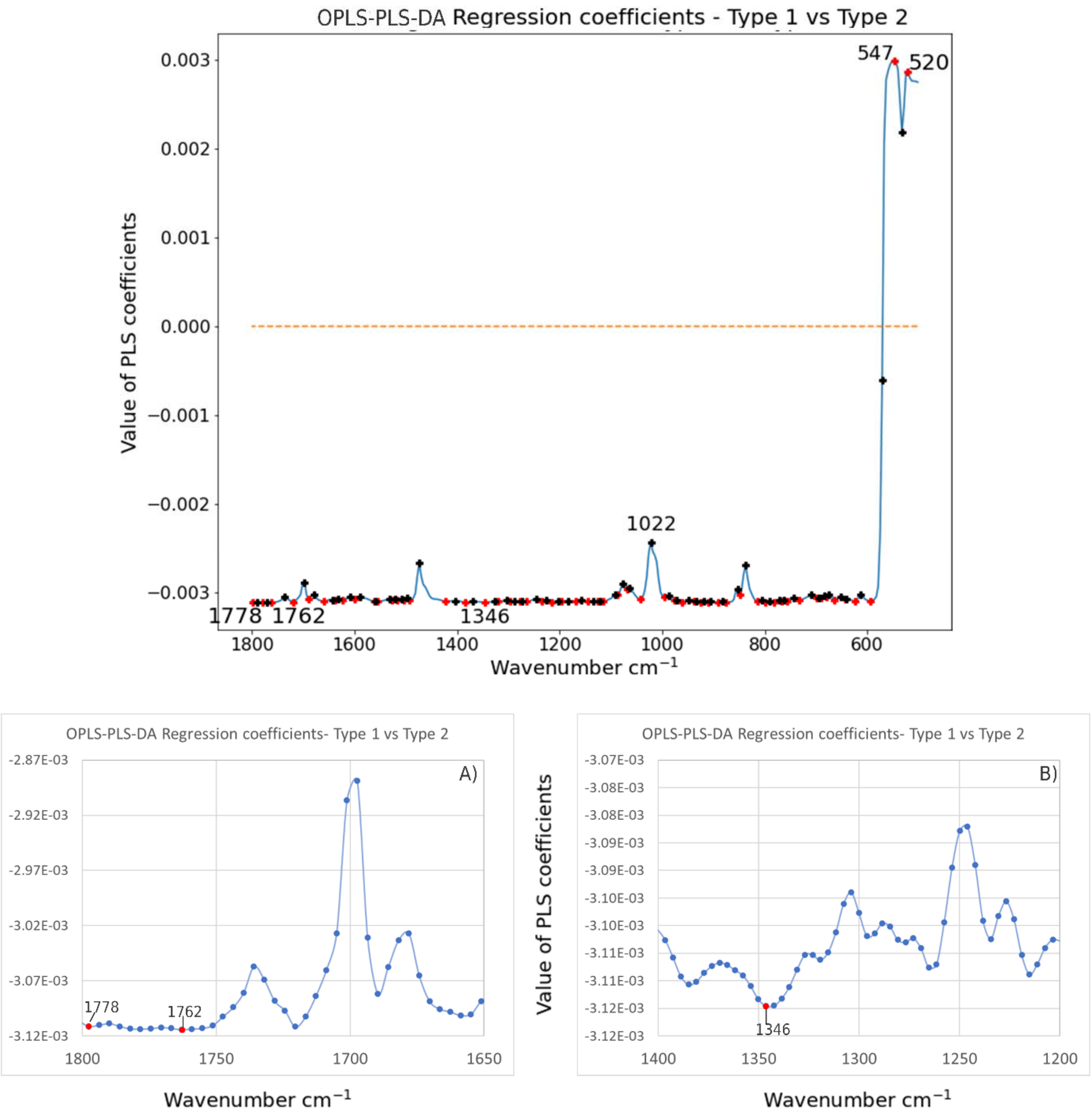


**Figure S6.** OPLS-PLS-DA Regression coefficients for Type 1 vs Type 2. A – B figures ilustrate a close-up to the regression coefficients regions where it is not possible to discern the highest coefficient scores in positive and negative leverage. Only the highest coefficient scores are shown.

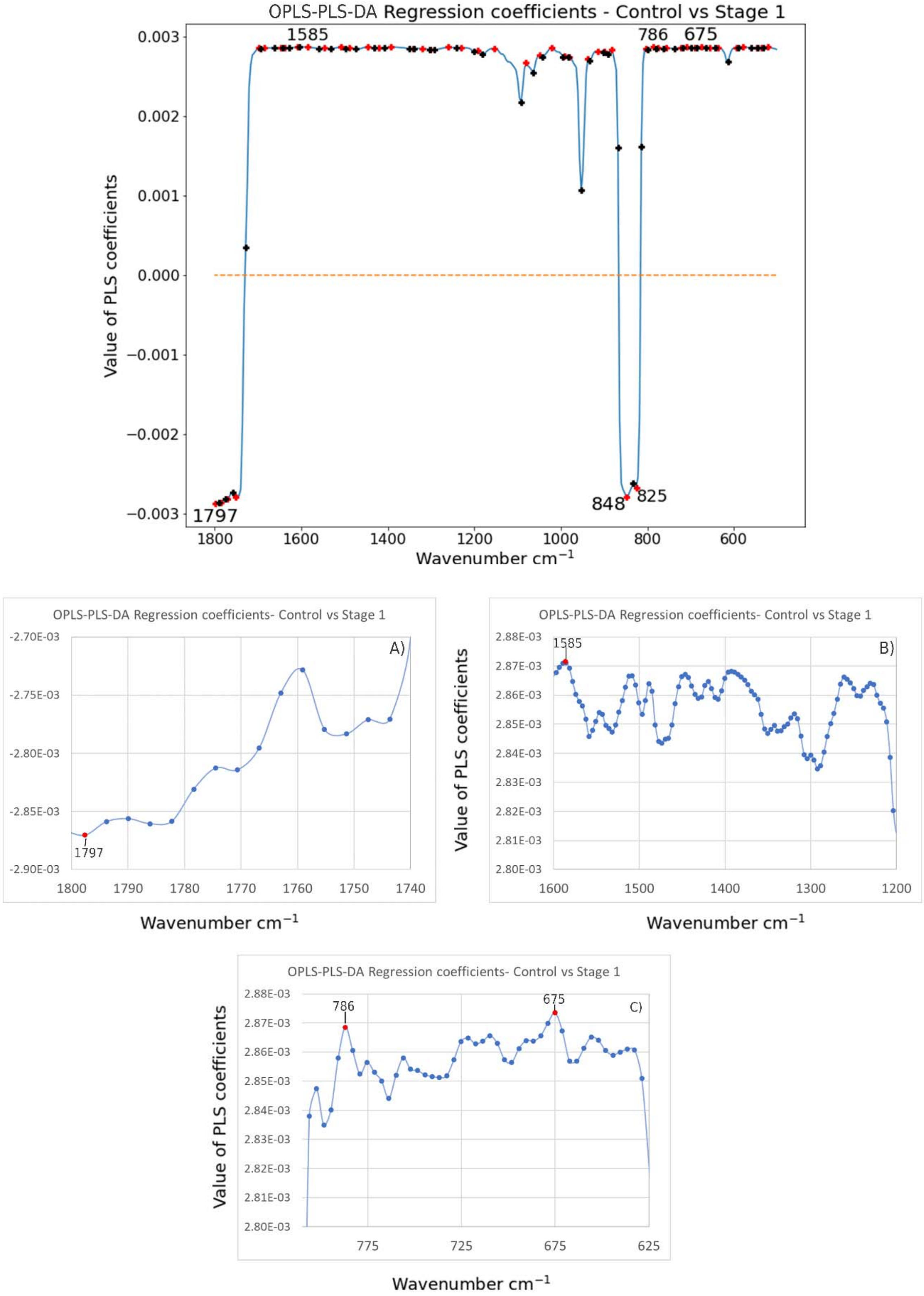


**Figure S7.** OPLS-PLS-DA Regression coefficients for Control vs Stage 1. A – C figures ilustrate a close-up to the regression coefficients regions where it is not possible to discern the highest coefficient scores in positive and negative leverage. Only the highest coefficient scores are shown.

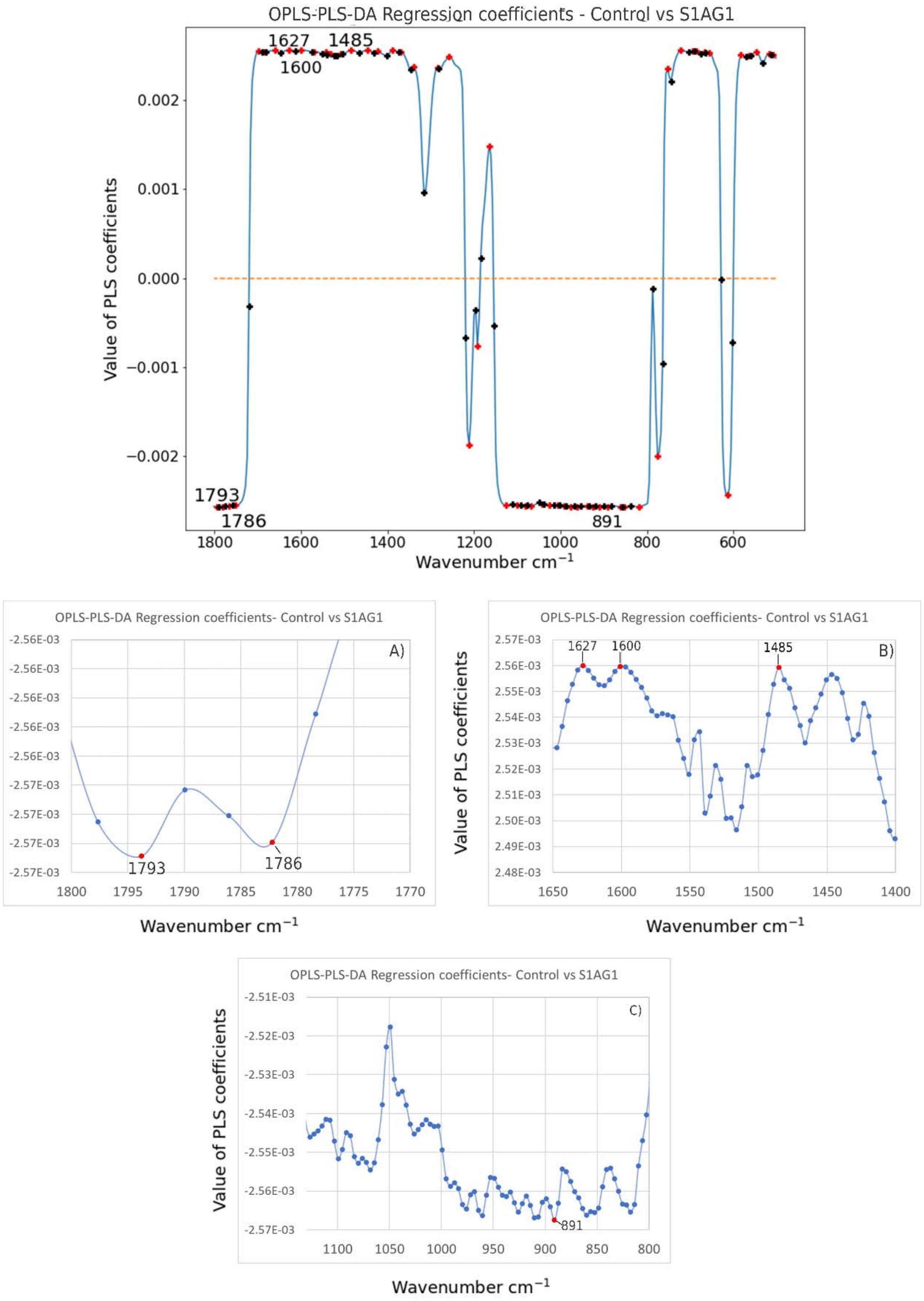


**Figure S8.** OPLS-PLS-DA Regression coefficients for Control vs S1AG1. A – C figures ilustrate a close-up to the regression coefficients regions where it is not possible to discern the highest coefficient scores in positive and negative leverage. Only the highest coefficient scores are shown.

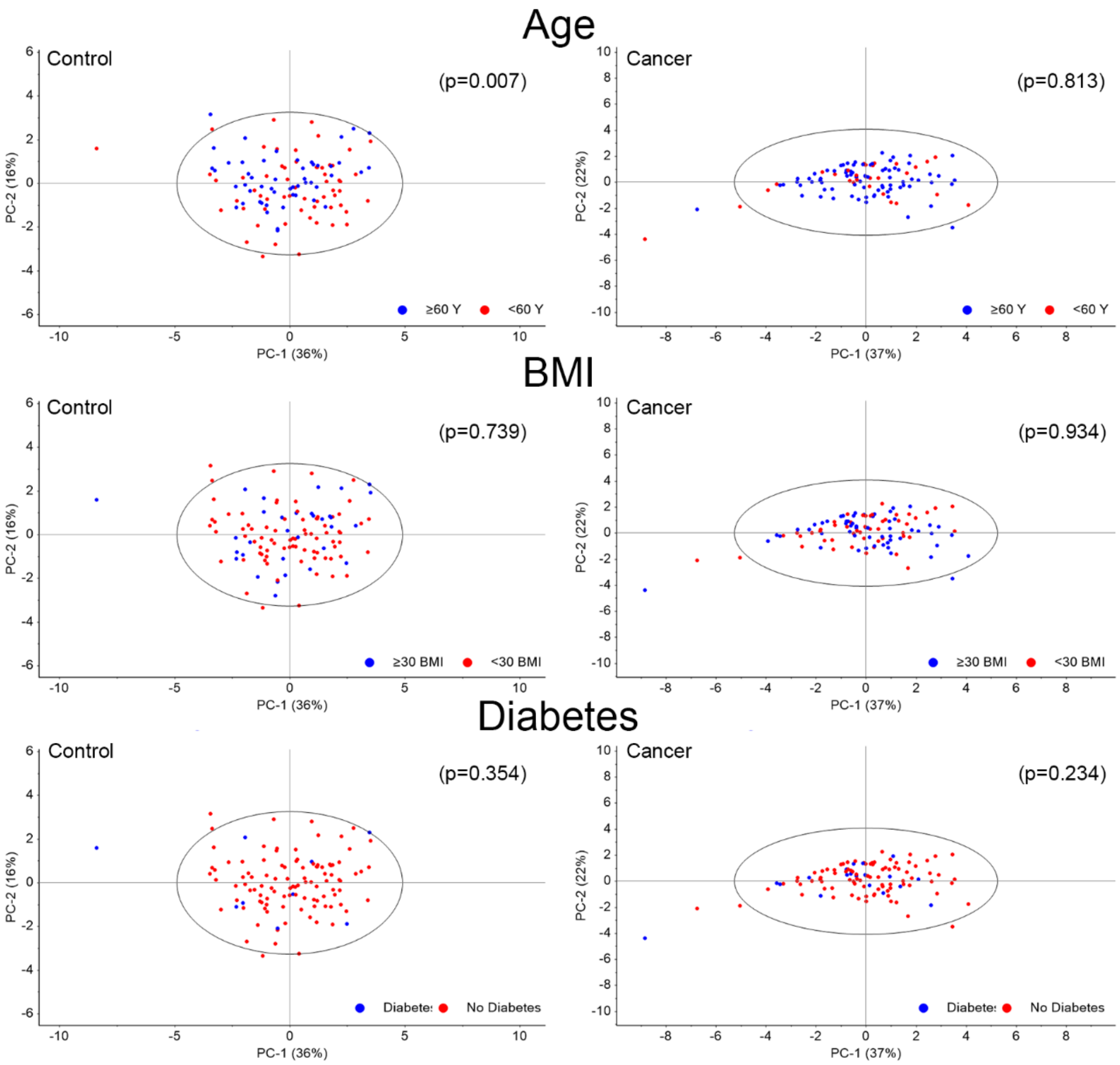


**Figure S9.** PCA score plots. These plots allow the visualisation of the confounding factors, by age, BMI, and diabetic status. Although the only group with a p-value of p<0.007 (control group by age, top plots) that shows statistical significance, it does not present any dramatic differentiation in the clusters. Furthermore, no comparisons are shown to be relevant according to the confounding factors.

**Table S1.** Binary predictions for all comparisons. Three major supervised models for the prediction were employed and compared between each other; PLS, KNN, SVC. Five different metrics were obtained to allow an adequate comparison; Sensitivity, specificity, precision, F1 score, and accuracy of prediction.

| | | | | | |
|---|---|---|---|---|---|
| | | | **Control vs. Cancer** | | |
| | **Sensitivity** | **Specificity** | **Precision** | **F1 Score** | **Accuracy of Prediction** |
| **PLS** | 83% | 93% | 88% | 88% | 88.095% (+/- 8.11%) |
| **KNN** | 87% | 91% | 89% | 89% | 89.50% (+/- 6.44%) |
| **SVC** | 84% | 94% | 90% | 89% | 88.10% (+/- 5.45%) |
| | | | **Control vs. Type 1** | | |
| | **Sensitivity** | **Specificity** | **Precision** | **F1 Score** | **Accuracy of Prediction** |
| **PLS** | 100% | 90% | 94% | 94% | 97.64 (+/- 1.36%) |
| **KNN** | 85% | 100% | 96% | 94% | 94.52% (+/- 5.09%) |
| **SVC** | 15% | 87% | 52% | 47% | 59.82 (+/- 7.51%) |
| | | | **Control vs. Type 2** | | |
| | **Sensitivity** | **Specificity** | **Precision** | **F1 Score** | **Accuracy of Prediction** |
| **PLS** | 86% | 100% | 94% | 94% | 95.22 % (+/- 3.78%) |
| **KNN** | 82% | 100% | 96% | 93% | 93.31% (+/- 4.95%) |
| **SVC** | 29% | 81% | 57% | 55% | 62.65% (+/- 9.71%) |
| | | | **Type 1 vs. Type 2** | | |
| | **Sensitivity** | **Specificity** | **Precision** | **F1 Score** | **Accuracy of Prediction** |
| **PLS** | 60% | 100% | 86% | 79% | 71.63% (+/- 18.29%) |
| **KNN** | 100 | 100 | 100 | 100 | 100% (+/- 0%) |
| **SVC** | 67% | 43% | 55% | 51% | 61.82% (+/- 12.03%) |
| | | | **Control vs. Stage 1** | | |
| | **Sensitivity** | **Specificity** | **Precision** | **F1 Score** | **Accuracy of Prediction** |
| **PLS** | 83% | 100% | 96% | 93% | 90.78% (+/- 6.95%) |
| **KNN** | 84% | 100% | 94% | 93% | 91.87% (+/- 6.15%) |
| **SVC** | 84% | 100% | 94% | 93% | 92.43% (+/- 4.33%) |
| | | | **Control vs. S1AG1** | | |
| | **Sensitivity** | **Specificity** | **Precision** | **F1 Score** | **Accuracy of Prediction** |
| **PLS** | 100% | 100% | 100% | 100% | 100% (+/- 0%) |
| **KNN** | 80% | 100% | 99% | 94% | 99.23% (+/- 0%) |
| **SVC** | 80% | 100% | 99% | 94% | 99.23% (+/- 0%) |

**Table S2.** Assigned bands for prospective biomarkers identified using PLS regression coefficients in all class comparisons.

| ***Wavenumber ($cm^{-1}$)*** | ***Assignment*** | ***Reference*** |
|---|---|---|
| *509* | Unassigned | - |
| *520* | Cα = Cα′ torsion and ring torsion of phenyl | [36,37] |
| *547* | Unassigned | - |
| *609* | Ring deformation of phenyl | [36,37] |
| *628*<br>*675*<br>*686*<br>*763*<br>*786* | CH out of plane bending vibration | [36,37] |
| *810* | Ring CH deformation | [36,37] |
| *~825* | C2′ endo conformation of sugar | [36] |
| *~848* | DNA | [36] |
| *891*<br>*964*<br>*1022* | Deoxyribose (C-C, C-O), DNA | [36,37] |

| | | |
|---|---|---|
| *1114* | Symmetric stretching P-O-C | [36] |
| *1338* | $CH_2$ wagging of collagen | [36] |
| *1346* | Amide III | [36] |
| *1419* | Deformation C-H | [36] |
| *1450* | $CH_3$ asymmetric bending of proteins | [36,37] |
| *1485* | CH deformation | [36] |
| *1585* | Ring deformation of phenyl | [36] |
| *1600* *1627* *1662* | Amide I | [36] |
| *1770* *1762* *1778* *1782* *1793* *1797* | Lipids (C=O, C=C stretching) | [36,37] |
